# Combined fluorescent *in situ* hybridization and F-*ara*-EdU staining on whole mount *Hymenolepis diminuta*

**DOI:** 10.1093/biomethods/bpaf011

**Published:** 2025-02-13

**Authors:** Mohamed Ishan, Isabell R Skipper, Tania Rozario

**Affiliations:** Department of Genetics and Center for Tropical & Emerging Global Diseases, University of Georgia, Athens, GA, United States; Department of Pharmacology and Chemical Biology, Emory University School of Medicine, Atlanta, GA, United States; Department of Genetics and Center for Tropical & Emerging Global Diseases, University of Georgia, Athens, GA, United States; Department of Genetics and Center for Tropical & Emerging Global Diseases, University of Georgia, Athens, GA, United States

**Keywords:** *Hymenolepis diminuta*, tapeworm, fluorescent *in situ* hybridization, F-*ara*-EdU staining, lineage tracing

## Abstract

*Hymenolepis diminuta* is a parasitic tapeworm that utilizes rats as hosts and offers advantages over human parasitic tapeworms and free-living flatworms as a model system to study the biology and pathology of helminth infections. *H. diminuta* is minimally infectious to humans, easy to maintain in the lab, demonstrates impressive growth, regeneration, and reproductive capabilities, and is amenable to loss-of-function manipulations. As an emerging model, tool development is critical to increasing the utility of this system. This study introduces a novel protocol for *H. diminuta* that combines fluorescent in situ hybridization (FISH) and 2′-Deoxy-2′-fluoro-5-ethynyluridine (F-*ara*-EdU) uptake and staining. Our protocol allows for the spatial detection of gene expression and simultaneous identification of proliferating cells. Dual labeling of F-*ara*-EdU and stem cell markers revealed a distinct expression pattern in different anatomical regions, especially in the head and neck. We demonstrate optimal labeling without permeabilization, streamlining the protocol. We also demonstrate generalizability using FISH for other tissue markers. The protocol was applied to perform bulk lineage tracing, revealing that stem cells can differentiate into neuronal and tegumental cells within 3 days. Our protocol provides an important tool in the arsenal for investigating gene expression and cell proliferation in *H. diminuta*, contributing valuable insights into the biology of parasitic tapeworms and potentially opening new avenues for the study of human parasitic tapeworms.

## Introduction

Parasitic flatworms, such as *Schistosoma mansoni* [1, 2] and tapeworms, such as *Taenia solium* [3], use humans as hosts, causing a detrimental impact on human health [4, 5]. Adult tapeworms have been notoriously difficult to study at the molecular level owing to the difficulty in maintaining their complex life cycles. *Hymenolepis diminuta* or the rat tapeworm has been (re)-established as a tractable model system [6], which is easily maintained in the lab. Not only is it poorly infectious to humans and thus safe to work with, but it also shares similarities with human parasitic tapeworms, such as rapid growth and regeneration [7]. Similar to most tapeworms, *H. diminuta* has a multi-host life cycle. Adult tapeworms develop reproductively mature hermaphroditic proglottids (apparent segments) that become gravid with eggs within the rat intestine. These gravid proglottids are shed with rat feces and can be consumed by a variety of intermediate host insects, such as beetles. Larval development occurs within the insect host until infective cysticercoids form and can remain dormant in their host. Rats that consume infected beetles or cysticercoids directly (in laboratory settings) complete the life cycle. Furthermore, *H. diminuta* can be grown *in vitro* and is amenable to gene knockdown by RNA interference (RNAi) making it a powerful model for understanding genotype–phenotype relationships in tapeworms [7].

One of the factors enabling *H. diminuta* to achieve rapid growth and regeneration is the presence of adult stem cells [7, 8]. These stem cells exhibit similarity to neoblasts, a stem cell population found in free-living flatworms like planarians [9–11]. In all flatworms analyzed to date, neoblast-like stem cells are the only dividing somatic cells that give rise to all differentiated cells [9, 12–15]. Understanding the role of this neoblast-like stem cell population in *H. diminuta* is crucial, as it provides insight into stem cell potency and plasticity, differentiation into tissue types important for parasite physiology, the formation of proglottids, the prolific rates of germ cell production in tapeworms, and many other processes that underlie the success and transmission of tapeworms.

As an emerging model organism, the development of modern tools and techniques is crucial to making meaningful discoveries. In this study, we developed a protocol that combines fluorescent *in situ* hybridization (FISH) [16, 17], to detect the spatial distribution of gene expression and 2′-Deoxy-2′-fluoro-5-ethynyluridine (F-*ara*-EdU) uptake and staining [18] to label proliferating cells in the synthesis phase of the cell cycle. This combined protocol enables us to pinpoint cells that express a particular gene of interest and are also currently undergoing proliferation. Hence, we will be able to characterize genes expressed within the stem cell population that might play important roles in parasite growth and regeneration. As adult *H. diminuta* can be cultured *in vitro*, we can also perform pulse-chase experiments to label the progeny of stem cells. Our protocol will enable the discovery of genes involved in stem cell differentiation and to characterize stepwise lineage relationships for genes of interest. The utility of simultaneously labeling proliferation-competent cells with marker gene expression is vast. Thus, it is an important technical advance in any organism that depends on stem cells for its survival and fecundity like *H. diminuta* and other flatworms.

## Materials and methods

### Animal use

Animal use was approved by the Institutional Animal Care and Use Committee at the University of Georgia (A2023 10-019-Y1-A0) and adhered to the National Institutes of Health guidelines for the care and use of animals in research. Sprague-Dawley rats were housed in the animal facilities within the Paul D. Coverdell Center for Biomedical and Health Sciences at the University of Georgia.

### Adult *H. diminuta* collection

During infection of rats with *H. diminuta* cysticercoids, sterile conditions were maintained, and facial protection was employed to avoid accidental consumption of cysticercoids. One-month infected *Tenebrio molitor* beetles were used. Cysticercoids were released from the beetle abdominal cavity into 0.85% NaCl and washed twice. Rats were infected by oral gavage with 100–350 cysticercoids aspirated into a 0.5% BSA-coated gavage needle in a maximum volume of 0.5 ml.

We typically collected adult tapeworms 6 days post-infection. At this stage, most tapeworms had visible gonads, but gamete production was minimal. Rats were humanely euthanized, and the small intestine was removed. Tapeworms were flushed out with Hanks Balanced Salt Solution (HBSS; Corning). Worms were cleaned by repeatedly moving them through the air–water interface with a stainless-steel hook (Moody Tools) in Working Hanks 4 (WH4: HBSS/4 g/l glucose/1X antibiotic–antimycotic).

### *In vitro* culture of tapeworms

Tapeworms were grown *in vitro* using biphasic parasite cultures modified from the Schiller method [19]. The solid phase was prepared by mixing 30% heat-inactivated defibrinated sheep blood (Hemostat) with 70% blood agar base (22.9 g/l BD Difco nutrient agar/5 g/l sodium chloride). Autoclaved blood agar base was stored at 4°C, melted, and cooled to 40–50°C before mixing with warmed blood. 10 ml of the solid phase mixture was aliquoted into 50 ml Erlenmeyer flasks and allowed to solidify for at least 10 min. 10 ml of WH4 was added, each flask topped with a sterile gas permeable stopper (Jaece identi-plug) and incubated at 37°C in hypoxia (5% CO_2_/5% O_2_/95% N_2_) overnight or for a minimum of 4 h. Before use, the liquid phase was adjusted to pH 7.5 with 300 µl 7.5% sodium bicarbonate (Corning). Tapeworms were transferred into flasks using stainless-steel hooks and immersed into the liquid phase. Incubations were performed in hypoxia, and worms were transferred into fresh cultures every 3–4 days as needed. For testing dimethyl sulfoxide (DMSO) concentrations, tapeworm fragments were grown in six-well plate format without agar to enable more accurate DMSO concentration titrations. The parasite cultures were prepared as described above, the liquid phase was poured into a conical, 4 ml aliquots were transferred to each well, and the desired volume of DMSO was added after removing the same volume of liquid culture. High-quality DMSO (Hybri-Max) was used for any application where DMSO was added to live tapeworms. DMSO stocks were aliquoted and frozen at −80°C and repeated freeze–thawing was minimized.

### F-*ara*-EdU treatment and fixation

Tapeworms were incubated in 0.1 μM F-*ara*-EdU/1% DMSO/WH4 at 37°C for 1 h unless otherwise stated. For pulse experiments, worms were washed 3 times in WH4 before immediate fixation. For pulse-chase experiments, worms were cultured *in vitro* for 3 days as described above. Worms were cleaned into WH4 before fixation.

For fixation, worms were transferred into glass scintillation vials in batches of 20 to avoid overcrowding, heat-killed in autoclaved deionized water at ∼80°C and immediately fixed in 10 ml FND fixative (4% formaldehyde/10% DMSO/1% NP40/PBSTx-DEPC (1× Phosphate Buffered Saline with 0.3% Triton X-100 pretreated with 0.1 M diethyl pyrocarbonate)). Fixation was limited to 30 min at room temperature followed by three washes in PBSTx-DEPC and dehydration into methanol. Worms were stored at −30°C for at least 2 days prior to use.

### Riboprobe synthesis

Antisense riboprobes were synthesized *in vitro* from ∼1 kb fragments of tapeworm cDNA cloned into vector pJC53.2 [20]. T3 or SP6 RNA polymerase was used for in vitro transcription depending on the direction of insertion (verified by Sanger sequencing). The PCR product was amplified with T7 primers that flanked the region of interest and cleaned with a DNA clean and concentrate kit (Zymo). *In vitro* transcription was performed overnight at 31°C in the following mixture: 13 µl clean PCR product, 2 µl RNA polymerase (made in-house), 1 µl RNAse inhibitor (Promega), 2 µl DIG-RNA labeling mix (10 mM ATP, CTP, GTP/6.5 mM UTP/3.5 mM DIG-11-UTP (Roche)/DEPC-water), 2 µl 10X transcription buffer (SP6 buffer: 0.4 M Tris pH8.0/0.1 M magnesium chloride/10 mM spermidine/0.1 M DTT or T3 buffer: 0.3 M HEPES pH7.5/1 M potassium glutamate/0.15 M magnesium acetate/2.5 mM EDTA/10 mM DTT/0.5% Tween-20). DNase (Promega) treatment was performed for 15 min at 37°C prior to RNA precipitation in 2.5 µl 4 M lithium chloride and 75 µl 100% ethanol at −30°C for at least 30 min. Riboprobes were precipitated by centrifuging at 18,000 RCF for 15 min at 4°C, pellets were washed in 70% ethanol twice, briefly air-dried, and resuspended in 50 µl DEPC-water. Riboprobe quality was verified by gel electrophoresis and quantified using a nanodrop spectrophotometer. Riboprobes were then diluted to ∼50 ng/µl in hybridization buffer (50% deionized formamide/5X SSC (Simple Sodium Citrate from a 20X stock of 0.3 M sodium citrate/3 M sodium chloride)/0.1 mg/ml yeast RNA (Invitrogen)/1% Tween-20/5% dextran sulfate/DEPC-water) and stored at −30°C. Riboprobes used in this study are listed in Supplementary Table S2.

### *In situ* hybridization

Our protocol was modified from previously published methods for planarians [21] and the mouse bile-duct tapeworm [22]. *H. diminuta* worms were gradually rehydrated into PBSTx-DEPC and treated with Proteinase K solution (10 μg/ml Proteinase K/0.1% SDS/PBSTx-DEPC) with gentle agitation for 30 min. Three washes in 10 ml of DEPC-treated 0.1 M triethanolamine hydrochloride (pH 7–8) were performed then 25 μl of acetic anhydride was added twice, 5 min apart while swirling to ensure complete dissolution. Two 5-min washes in PBSTx-DEPC were followed by post-fixation in 4% formaldehyde/PBSTx-DEPC for 10 min at room temperature. After three washes in PBSTx-DEPC, worms were transferred to medium incubation baskets with 100 µm mesh bottoms (CEM) in a 24-well plate. Worms were equilibrated in equal parts PBSTx-DEPC and prehybridization buffer (hybridization buffer without dextran sulfate; refer above) for 10 min. Prewarmed prehybridization buffer was added and worms were incubated at 56°C for 2 h. Transcript-specific riboprobe mixes were prepared by heating aliquots of each riboprobe in a thermocycler at 80°C for 10 min, cooling on ice, then diluting in pre-warmed hybridization buffer at 1:1000 from stocks of 50 ng/μl. Worms were incubated in riboprobe mix at 56°C overnight (2-day incubation can be applied for a stronger signal if necessary). After riboprobe hybridization, all solutions used were not DEPC-treated. Stringent washes at 56°C were performed with two preheated wash solutions: 2X- and 0.2X-SSCx (SSC supplemented with 0.1% Triton X-100). First, half of the riboprobe mix was removed and replaced with an equal volume of 2X SSCx to equilibrate for 20 min. The worms were then washed twice in 2X SSCx and 4 times in 0.2X SSCx at 56°C for 20 min per wash. Two washes in TNTx (0.1 M Tris pH7.5/0.15 M NaCl/0.3% Triton X-100) for 10 min each were performed then worms were blocked for 2 h in 10% horse serum/0.5% Roche Western Blocking Reagent (RWBR)/TNTx at room temperature. Overnight antibody incubation at 4°C was performed using anti-DIG-POD antibody (Roche) at 1:2000 in a blocking solution. Eight TNTx washes for 20 min were performed before signal development using Tyramide Signal Amplification (TSA). DL633 TSA development solution (1:250 DL633-tyramide made in-house from NHS ester/1: 1000 4-iodophenylboronic acid (4-IPBA, from 20 mg/ml stock)/0.003% H_2_O_2_/TSA buffer (2M sodium chloride/100 mM borate pH8.5)) was prepared. In-house tyramide-fluorophore conjugates were synthesized according to Ryan King’s adaptation, based on the method of Hopman *et al*. [23], previously published in King and Newmark [21]. TSA reaction was performed for 20 min. Four washes were performed in TNTx and peroxidase activity was inactivated in 100 mM sodium azide in PBSTx for 45 min.

### F-*ara*-EdU staining of *Hymenolepis diminuta*

Peroxidase-inactivated worms were washed in TNTx 4 times for 10 min each then treated with Proteinase K solution for 30 min (minimum 15 min) at room temperature without shaking. Post-fixation was performed in 4% formaldehyde/PBSTx for 10 min followed by three 5 min PBSTx washes. Small worms could be processed whole, while larger worms were cut into smaller pieces. An optional incubation in NDP permeabilization solution (1% NP40/10% DMSO/PBSTx) for 1 h at room temperature was conducted, followed by three washes in PBSTx to test if increased permeabilization was achieved. For the Click-iT reaction, worms were placed in 1.7 ml Eppendorf tubes to minimize the reaction volume; PBSTx was removed under a microscope before adding 200 μl of Click-iT reaction mix (0.1M PBS/1 mM copper sulfate/0.1 mM Oregon Green 488 azide/100 mM ascorbic acid, added in that order with mixing in between). Click-iT reaction can be performed in baskets in a minimum volume of 300 μl but must be scaled up with increasing tissue mass. The worms were incubated for 30 min at room temperature, with periodic gentle mixing. Worms were transferred to baskets if needed and washed 3 times in PBSTx for 10 min each. The worms were then blocked in K-block (5% horse serum/0.45% fish gelatin/0.3% Triton-X/0.05% Tween-20/0.1 M PBS) at room temperature for 2 h or overnight at 4°C. Anti-Oregon Green 488-HRP antibody (Invitrogen) was added at 1:1000 in K-block and incubated overnight at 4°C. The worms were washed 8 times in TNTx and TSA development was performed as described above but with 1:500 TAMRA-tyramide (made in-house from 5- (and -6)- carboxytetramethylrhodamine) for 20 min. Worms were washed 3 times for 10 min each in TNTx and stained with 1 µg/mL 4′,6-Diamidine-2′-phenylindole dihydrochloride (DAPI) in TNTx overnight at 4°C, followed by three additional washes for 5 min each in TNTx. If worms were too sticky or fragile, they were post-fixed and washed. The worms were removed from baskets and equilibrated in mounting solution (80% glycerol/10 mM Tris pH7.5/1 mM EDTA) for at least one night at room temperature. Worms were mounted on slides in mounting solution with #1.5 coverslips and sealed with nail polish.

### Imaging

Single-plane micrographs and z-stacks were captured with a 63× objective (Plan-Apochromat 63×/1.40 Oil DIC M27) with 0.35 µm plane thickness or a 20× objective (Plan-Apochromat 20×/0.8 M27/FWD = 0.55 mm) with 2 μm plane thickness. Imaging was performed on a laser scanning confocal microscope (Zeiss LSM 900). Only brightness/contrast adjustments were made using Fiji [24].

### Quantification

The density of F-*ara*-EdU^+^ cells was quantified using Imaris (Oxford Instruments). 3D confocal z-stacks were rotated and cropped to obtain a 500-µm-wide region of interest posterior to the scolex/head. Background subtraction was enabled, automatic spot discovery was applied, and then manually adjusted to label all visible spots without false positives. Snapshots of the projection were imported into Fiji to measure the worm area. Density of F-*ara*-EdU was calculated by dividing the number of positive cells over the area.

Colocalization quantification was performed on 63X confocal z-stacks in Fiji. Only the F-*ara*-EdU^+^ channel was made visible, cells were randomly selected and marked using the cell counter tool. Cells from two regions were selected: (i) internal tissues (up to 15 cells within the parenchymal region between the osmoregulatory canals and (ii) lateral tissues (up to 15 cells between a canal and the worm edge to encompass the nerves cords, tegument, and surrounding cells). For each marked cell, the DAPI and *in situ*^+^ channels were made visible to record if the cell was double^+^. For Fig. 6E, the same strategy was employed except that up to 20 *in situ*^+^ cells were selected randomly first and then the F-*ara*-EdU channel was made visible to record double^+^ cells. Two independent stainings were performed and 5–6 worms were imaged to obtain 14–17 z-stacks for every group at each timepoint.

### Statistical analysis

All statistical analyses were performed using Graph Pad Prism version 10 software. All experiments were repeated at least twice. Error bars, statistical tests, and *P*-values are indicated in corresponding figure legends (Supplementary Figs S1–S5 and Supplementary Tables S1 and S2).

## Results

Gene expression analysis by FISH and F-*ara*-EdU staining methodologies has been extensively employed in the examination of the *H. diminuta* tapeworm [7] and other systems. Despite the recognized effectiveness of each technique independently, there is currently a lack of a unified protocol integrating both FISH and F-*ara*-EdU for *H. diminuta*. Such an advance would enable more sophisticated analyses of cycling stem cells and their progeny.

**Figure 1.**
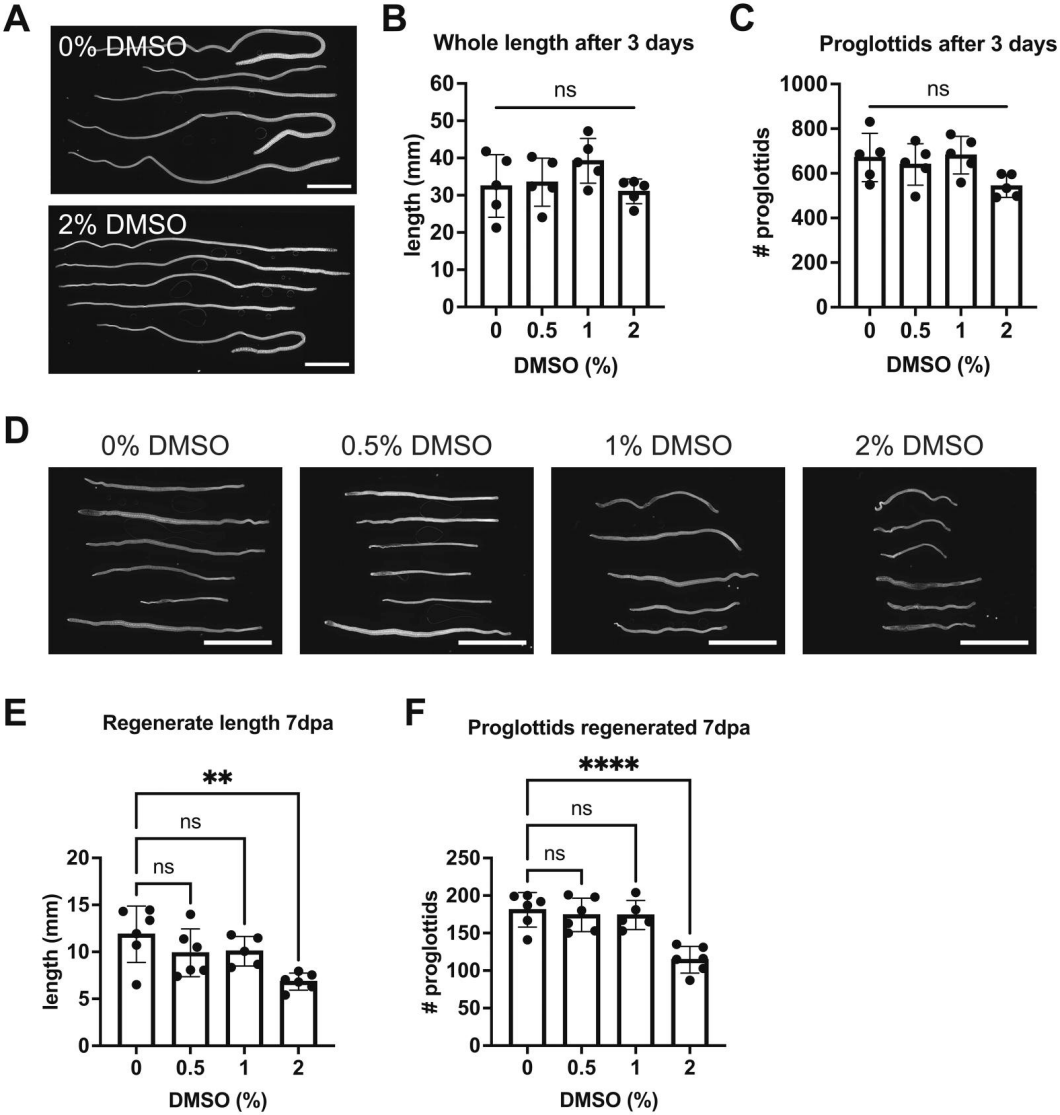
Effect of DMSO on tapeworm growth and regeneration. (A–C) Whole worms grown in DMSO for 3 days from a representative experiment. (D–F) Regenerates from 2 mm anterior fragments grown in DMSO for 7 days from a representative experiment. Gross morphology was examined using DAPI staining (A and D) with scale bars = 5 mm. Worm lengths (B and E) and the number of proglottids (C and F) were quantified and compared using one-way ANOVA compared to 0% DMSO with Dunnett’s multiple comparison testing; **P < .01, ****P < .0001, ns = not significant.

F-*ara*-EdU is administered in DMSO, so it was necessary to establish a safe concentration of DMSO for *H. diminuta*. To our surprise, *H. diminuta* is remarkably tolerant to high concentrations of DMSO. Continuous culture of whole 6-day-old worms in up to 2% DMSO for 3 days did not outwardly affect worm morphology or length (Fig. 1A and B). In 2% DMSO, the number of proglottids formed was decreased, though this was not statistically significant (Fig. 1C). As *H. diminuta* is competent to regenerate in a stem-cell-dependent manner [7], we tested if DMSO affects proglottid regeneration by amputating 2 mm anterior fragments from tapeworms that were first acclimated to *in vitro* culture conditions. After 7 days of continuous culture in 2% DMSO, there was increased cellular debris in the media and though the worms appear relatively normal (Fig. 1D), the lengths and number of proglottids regenerated were significantly reduced (Fig. 1E and F). However, continuous culture in up to 1% DMSO for 7 days had no discernable effects (Fig. 1D–F).

To determine the acceptable concentration of F-*ara*-EdU, we exposed tapeworms to a wide range of F-*ara*-EdU concentrations in 1% DMSO. No outward effects were observed when 6-day-old tapeworms were exposed to up to 100 µM F-*ara*-EdU for over 6 h. However, mature tapeworms (at least 2 weeks old) convulsed and contracted in 10 µM F-*ara*-EdU (data not shown). After staining, F-*ara*-EdU was detectable at concentrations as low as 0.01 µM but at low intensity. We conclude that 0.1–1 µM is a conservative range for F-*ara*-EdU uptake in *H. diminuta* though higher concentrations are likely appropriate for some applications.

**Figure 2.**
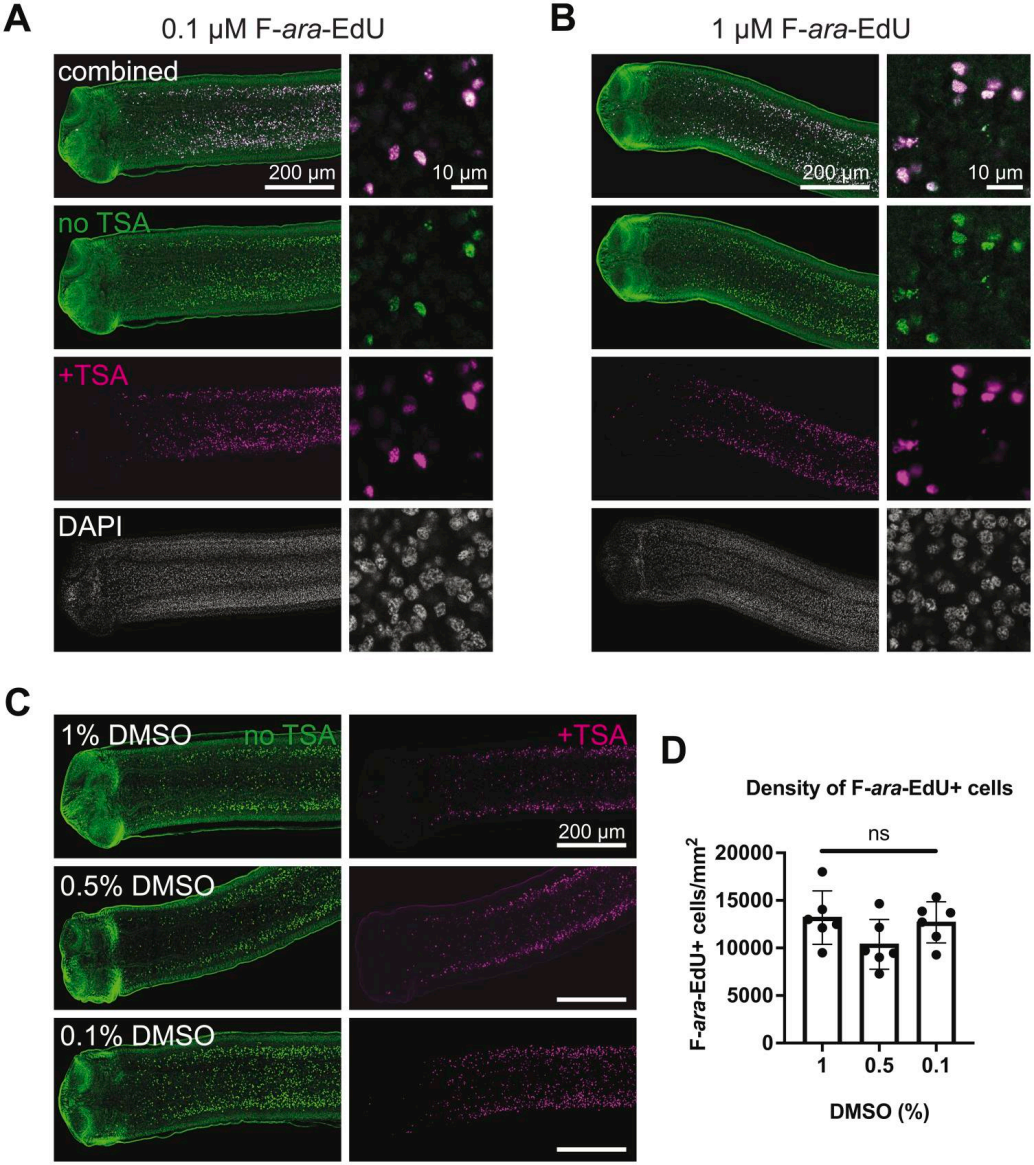
Efficacy of F-*ara*-EdU labeling following TSA reaction and exposure to different DMSO levels. (A–C) Single confocal micrographs at low and high magnification stained for F-*ara*-EdU without TSA (green), F-*ara*-EdU after TSA (magenta) and DAPI (gray). (D) Quantification of F-*ara*-EdU^+^ cell density from confocal z-stacks in a 500 µm wide region of the GR below the head normalized to area from a representative experiment. One-way ANOVA comparing all groups with Tukey’s multiple comparison testing; ns = not significant; error bars = SD.

F-*ara*-EdU is detectable using copper(I)-catalyzed azide-alkyne “click” chemistry [18]. We used Oregon-Green azide and were able to detect F-*ara*-EdU reliably at 0.1 µM (Fig. 2A). However, there was high background/noise at all concentrations tested. As Oregon-Green can be recognized by an antibody conjugated to horseradish peroxidase, TSA can be performed to increase the signal-to-noise ratio. This strategy has been effectively employed in planarians [21]. The addition of TSA greatly improved signal intensity while decreasing background, especially at low magnification (Fig. 2A and B). The background was not decreased significantly by increasing F-*ara*-EdU concentration (Fig. 2B). At high magnification, the background signal was still abundant but adjusting the brightness and contrast enabled acquisition of comparable images with or without TSA (Fig. 2A and B).

The high signal obtained after TSA led us to test if the amount of DMSO used can be lowered. Unfortunately, when less than 1% DMSO was used, F-*ara*-EdU penetration, signal intensity, and even background were inconsistent and unreliable. In experiments where good staining was observed at low DMSO concentrations, we quantified the density of proliferating cells in the germinative region (GR): the regeneration-competent tissue. No statistically significant difference in the density of F-*ara*-EdU was observed (Fig. 2C and D). Taken together, we conclude that 0.1 µM F-ara-EdU/1% DMSO is appropriate for labeling cycling cells in *H. diminuta*.

**Figure 3.**
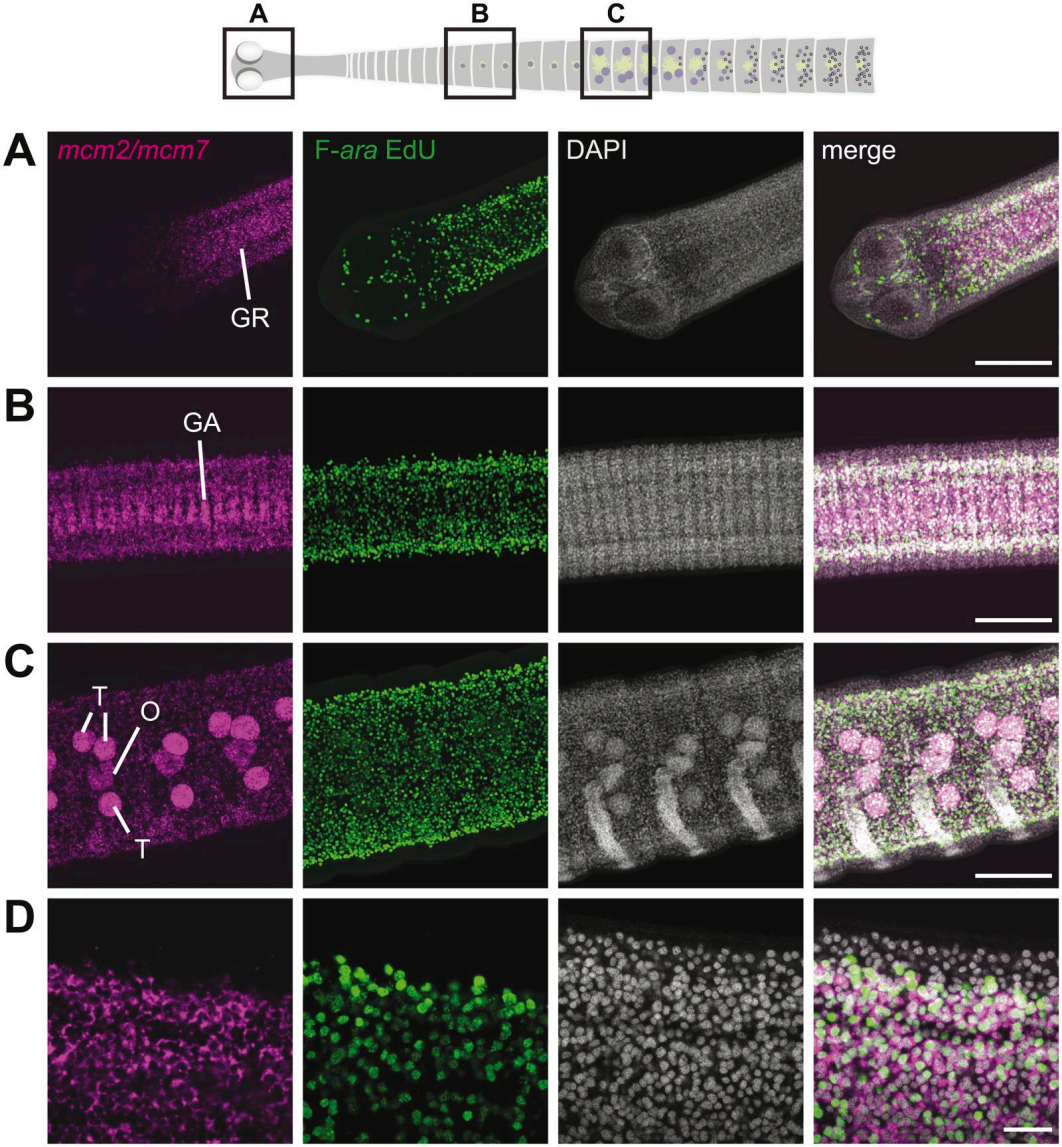
Co-localization of F-*ara*-EdU incorporation and cycling cell marker expression. Laser-scan confocal micrographs of *H. diminuta* stained by FISH for cycling cell markers *mcm2/mcm7* followed by detection of S-phase marker, F-*ara*-EdU . Nuclei are labeled with DAPI. Displayed regions include (A): head and neck/GR, (B): immature proglottids, (C): reproductive proglottids. (A–C) Represent maximum intensity projections, while (D) is a high-magnification single-plane confocal micrograph from the GR. GR: germinative region; GA: genital anlagen; T: testis and O: ovary. Scale bars: 100 µM in (A–C), 20 µM in (D).

To establish a combined protocol for FISH and F-*ara*-EdU uptake and staining, we used pooled antisense probes for cycling stem cell markers *minichromosome maintenance complex component-2* and -*7* (*mcm2/mcm7*) in 6-day-old *H. diminuta* tapeworms that were exposed to F-*ara*-EdU for 1 h at 37°C. Previous studies have demonstrated that limited exposure to F-*ara*-EdU (2 h) labels cycling cells within the parenchyma where *mcm2*^+^ stem cells reside [7]. If our protocol is successful, we expect to recapitulate this pattern and observe the co-occurrence of F-*ara*-EdU with *mcm2/mcm7*. When we stained for F-*ara*-EdU prior to performing FISH, neither signal was reliably detectable. However, performing FISH followed by F-*ara*-EdU staining was highly successful (Fig. 3). We were able to detect strong signals within the parenchyma of the regeneration-competent neck/GR (Fig. 3A), immature proglottids including the genital anlagen (GA: Fig. 3B) and in reproductive proglottids where gonadal development had begun (Fig. 3C). Our results recapitulate the expected patterns for singly stained worms for either F-*ara*-EdU or *mcm2* [7]. High magnification single plane confocal images confirmed that F-*ara*-EdU^+^ cells express *mcm2/mcm7* (Fig. 3D).

Tapeworms are surrounded by a tegument (skin) with limited permeability that has previously necessitated additional permeabilization steps with NP-40, DMSO, and Triton-X (NDP permeabilization) [7, 25]. We tested three permutations to identify optimal conditions: (i) proteinase K and NDP permeabilization (Fig. 3), (ii) proteinase K only (Supplementary Fig. S2), and (iii) NDP permeabilization only (Supplementary Fig. S3). Surprisingly, no significant differences in signals for either F-*ara*-EdU or *mcm2/mcm7* were observed among the three conditions. All conditions displayed good penetration, with labeled cells visible in deeper layers of the worm. However, NDP permeabilization occasionally produced suboptimal FISH and increased tissue fragility. Thus, we opted to omit the NDP permeabilization step for subsequent experiments. A detailed step-by-step protocol can be found in Supplementary Fig. S1.

**Figure 4.**
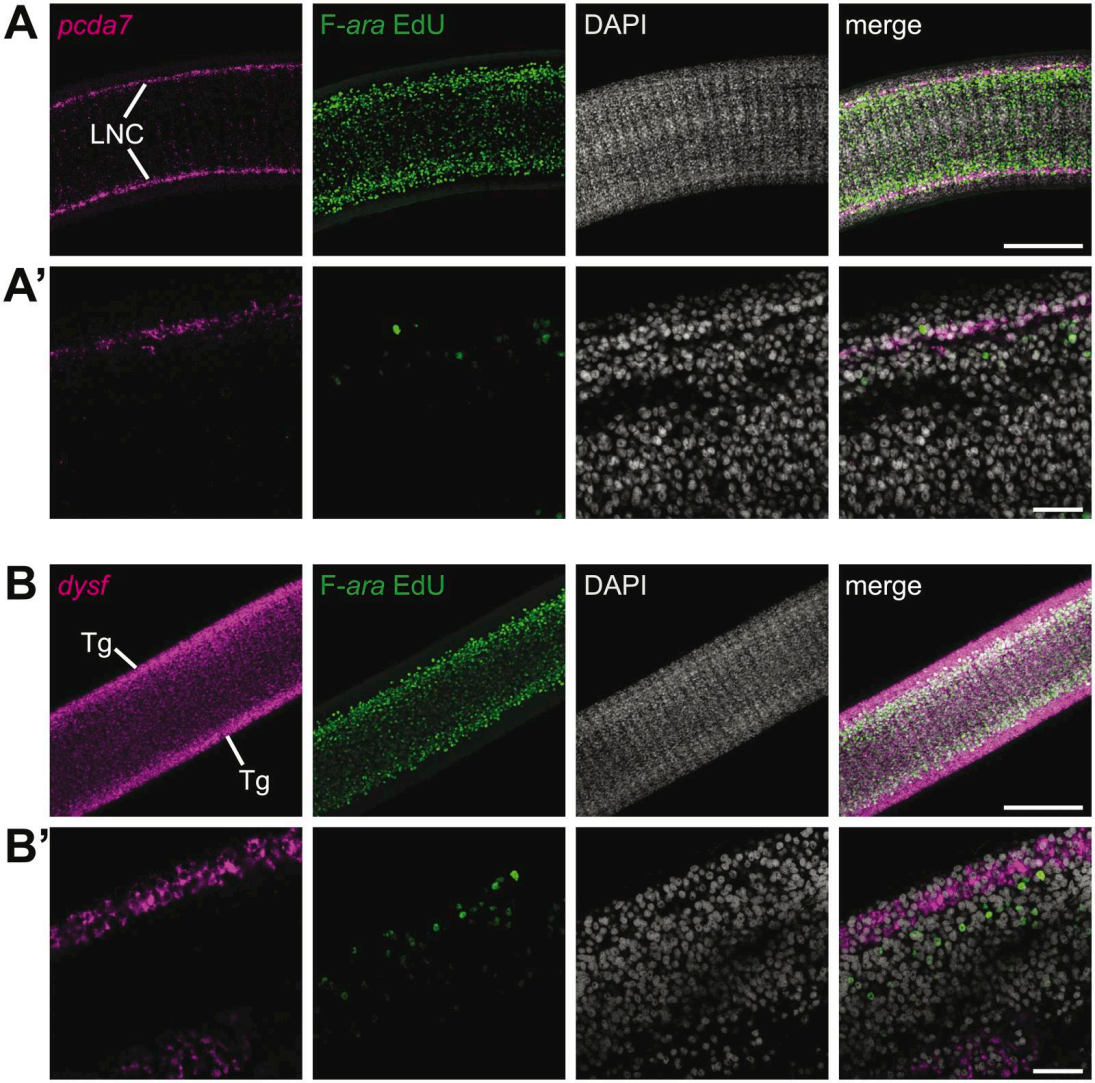
Combination protocol is effective in visualizing FISH of multiple transcripts with F-*ara*-EdU. Laser-scan confocal micrographs of *H. diminuta* stained by FISH for neuronal marker, *pcda7* (A–A′) and tegument marker, *dysf* (B–B′) followed by detection of S-phase marker, F-*ara*-EdU. Nuclei are labeled with DAPI. (A) and (B) represent maximum intensity projections through immature proglottids, while (A′) and (B′) show high-magnification single-plane confocal micrograph from the neck. LNC: lateral nerve cords and Tg: tegument. Scale bars: 100 µM in (A) and (B), 20 µM in (A′) and (B′).

To verify that our protocol could work with FISH for markers of other cell types, two additional riboprobes were tested: a neuronal marker, *protocadherin-alpha7* (*pcda7*), and a tegument marker, *dysferlin* (*dysf*). These markers were chosen because they are strongly expressed in differentiated tissues. Previously, *pcda7* was shown to be expressed in the nerve cords, longitudinal and transverse neural projections, and not in cycling stem cells [7]. We find that the lateral nerve cords (LNCs) strongly express *pcda7* while the F-ara-EdU^+^ cells are mostly contained within the LNC boundaries (Fig. 4A and A′). To label the tegument, we used *dysf*, which is expressed in the tegument of both *H. diminuta* and *S. mansoni* [26, 27]. Our protocol recapitulated *dysf* FISH at the outer surface surrounding the F-*ara*-EdU^+^ population within the interior parenchyma (Fig. 4B and B′). These results demonstrate that our protocol can be used for FISH with markers of differentiated tissues and is not riboprobe-specific.

**Figure 5.**
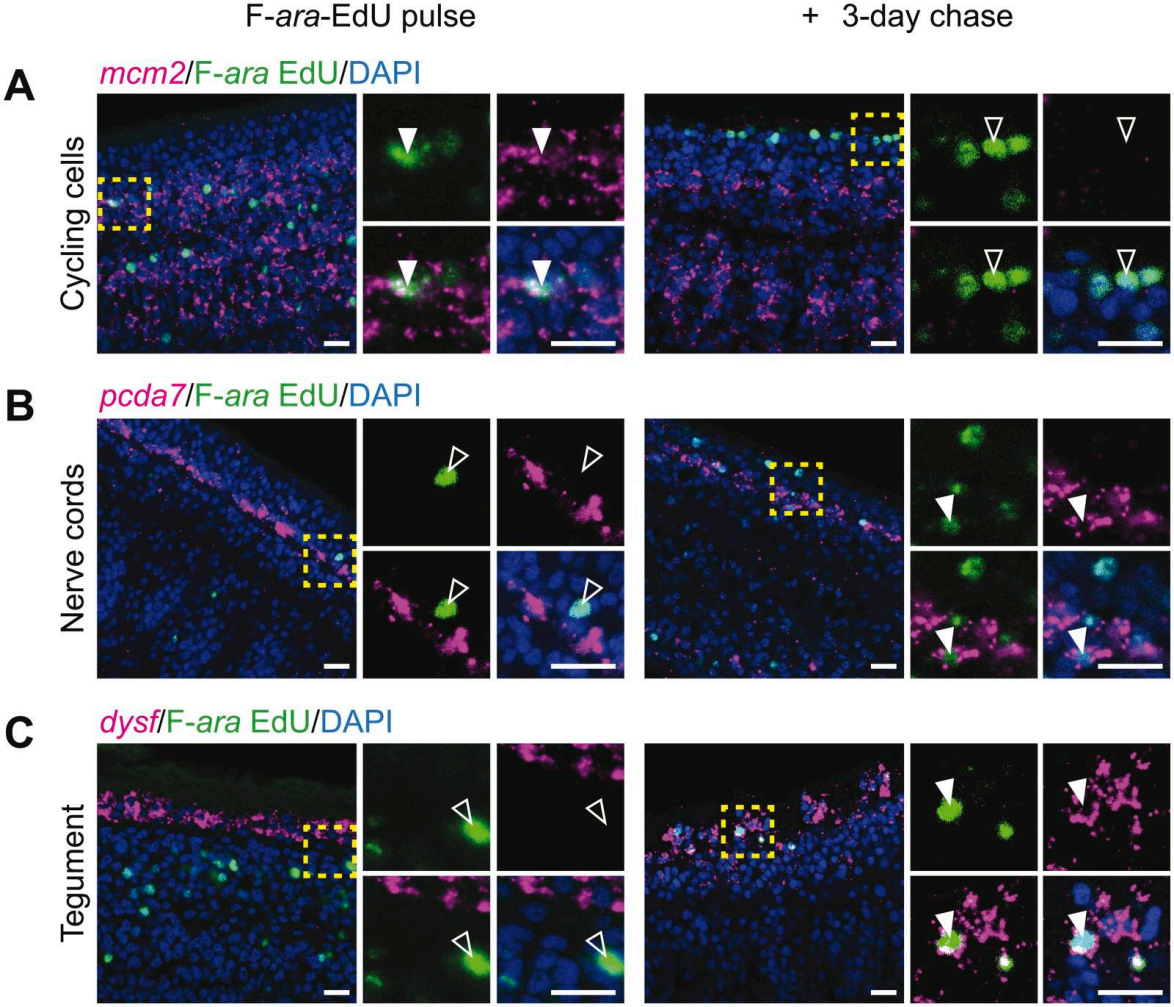
Combination protocol can be applied to bulk lineage tracing experiments. Single-plane confocal micrographs from necks of *H. diminuta* worms that were either pulsed with F-*ara*-EdU for 1 hr to label a subset of cycling cells and immediately fixed (left) or kept *in vitro* for 3 days to chase F-*ara*-EdU into differentiated progeny (right). FISH for *mcm2*, *pcda7* and *dysf* are shown. Nuclei are labeled with DAPI. Insets are outlined by the yellow boxes. Filled arrowheads point to incidences where marker gene expression and F-*ara*-EdU co-occur within cells whereas unfilled arrowheads point to incidences where marker gene expression and F-*ara*-EdU do not co-occur. Scale bars: 10 µM.

One utility of combination staining for F-*ara*-EdU and FISH is that it can be applied for bulk lineage tracing experiments. This is particularly powerful in the absence of transgenesis for lineage tracing in parasitic flatworms. If our protocol is successful, then we should be able to label only undifferentiated cycling stem cells following a short pulse of F-*ara*-EdU. On the other hand, a pulse of F-*ara*-EdU followed by extended *in vitro* culture should chase the thymidine analog into progeny that differentiated from the labeled stem cell pool. We successfully validated that our protocol can be applied for bulk lineage tracing using FISH makers for cycling stem cells, nerve cords and tegument (Fig. 5). After a 1 h pulse only, F-*ara*-EdU was detected within *mcm2*^+^ cycling cells (Fig. 5A; filled arrowheads) while differentiated cells expressing either *pcda7* or *dysf* were unlabeled with F-*ara*-EdU (Fig. 5B and C; unfilled arrowheads). After a 3-day chase period, F-*ara*-EdU was now detectable outside of the *mcm2*^+^ parenchymal space (Fig. 5A; unfilled arrowheads). We were also able to detect F-*ara*-EdU within differentiated cells expressing *pcda7* or *dysf* only after the 3-day chase (Fig. 5B and C; filled arrowheads).

**Figure 6.**
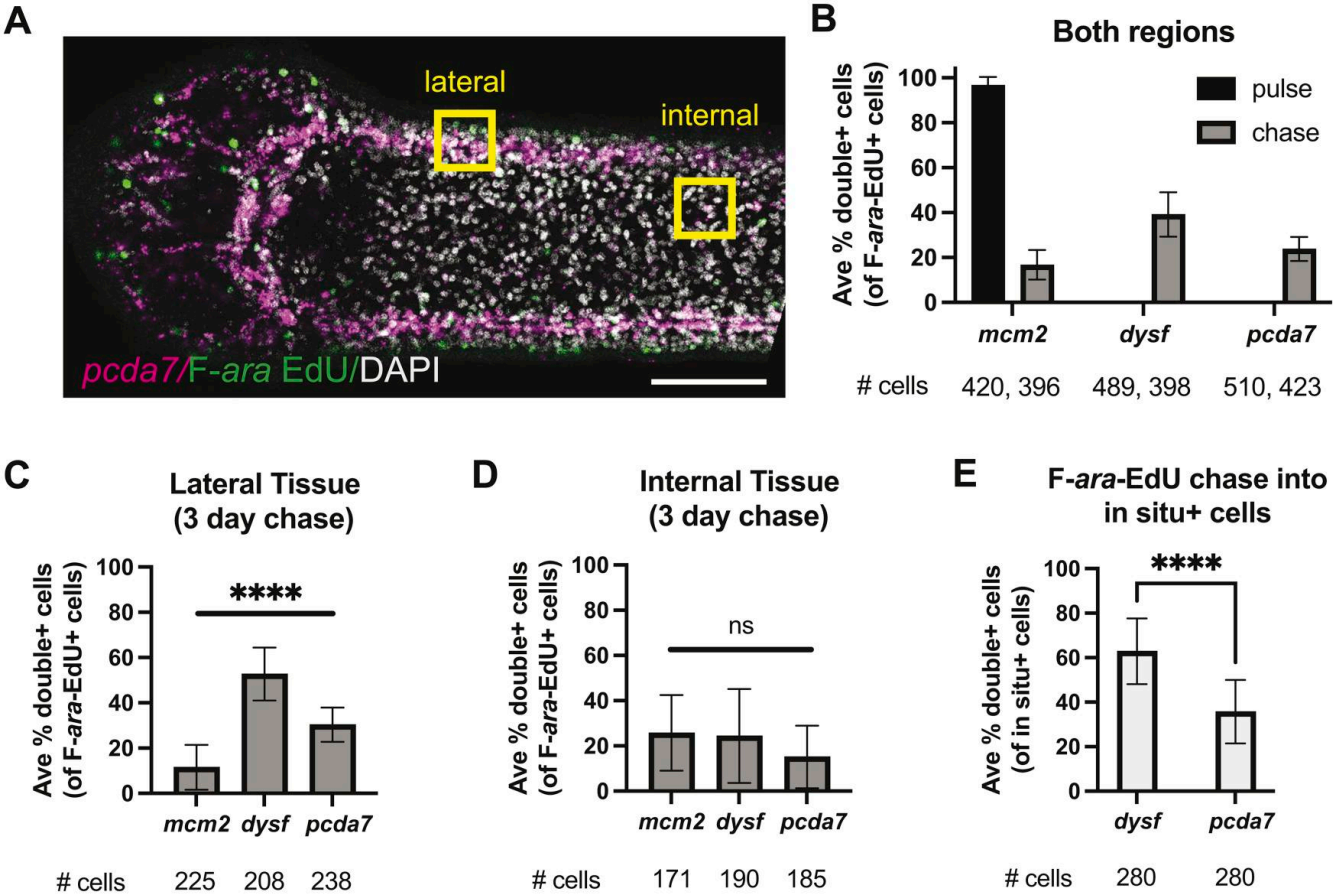
Quantification of bulk lineage tracing into tegumental and neuronal fates. (A) Single-plane confocal micrograph after 3-day pulse-chase labeled for *pcda7*, F-*ara*-EdU and DAPI-. Yellow boxes indicate approximate areas used for quantification in (B–E). (B) Average double^+^ cells after F-*ara*-EdU pulse or 3-day chase as a percentage of F-*ara*-EdU^+^ cells from both lateral and internal tissues. The number of F-*ara*-EdU^+^ cells counted are indicated under the *X*-axis (pulse, chase). (C and D) Average double^+^ cells after 3-day chase as a percentage of F-*ara*-EdU^+^ cells from lateral (C) or internal (D) tissues. All groups compared to each other using one-way ANOVA with Tukey’s multiple comparison correction. (E) Average double^+^ cells after 3-day chase as a percentage of *in situ*^+^ cells from lateral tissues only. Student’s *t*-test performed. For all: error bars = SD; ns = not significant, ****P < .0001. Example confocal micrographs used for quantification can be found in Supplementary Fig. S4 and S5.

To capture the dynamics of stem cell differentiation, we quantified co-occurrence of F-*ara*-EdU and *in situ* markers in pulse and pulse-chase experiments. Confocal z-stacks were acquired from two regions: lateral tissues where differentiated tegument and nerve cords would be readily observed and internal tissues that contain a mixture of many different cell types including a large proportion of stem cells (Fig. 6A). After an F-*ara*-EdU pulse, 97 ± 4% of F-*ara*-EdU^+^ cells expressed the stem cell marker *mcm2*, whereas 0% expressed either *dysf* or *pcda7* (Fig. 6B). We expected to find *dysf*^+^ cells at the edge most surface [28] as well as within tegumental progenitors as has been reported for *S. mansoni* [27]. We readily detected *dysf*  ^+^ cells in the lateral and internal tissues though none of them co-labeled with F-*ara*-EdU after the pulse (Supplementary Fig. S4). The same pattern was observed for *pcda7*^+^ cells; after the F-*ara*-EdU pulse, no co-labeled cells were detected at the lateral tissues containing the nerve cords or in the internal tissues where fine neural projections extend in both longitudinal and transverse commissures (Supplementary Fig. S4). After a 3-day chase period, the co-occurrence of F-*ara*-EdU and *mcm2* decreased to 17 ± 6% (Fig. 6B). At the same time, *dysf*^+^ and *pcda7*^+^ cells arose from the labeled stem cell pool (Fig. 6B). We were able to detect FISH signal in both lateral and internal tissues that coincided with F-*ara*-EdU after the 3-day chase period (Supplementary Fig. S5). The most significant changes were seen in the lateral tissues where 53 ± 12% F-*ara*-EdU^+^ cells expressed *dysf*, while 15 ± 14% F-*ara*-EdU^+^ cells expressed *pcda7* (Fig. 6C). Co-occurrence was also detected in the internal tissues though to lesser extents (Fig. 6D).

Though statistically significant differences for F-*ara*-EdU chase into the two tissue types were observed, this could be explained by unequal abundance or distribution of marker gene expression. Furthermore, detection of F-*ara*-EdU^+^/*pcda7*^+^ cells was highly variable between samples, which would be expected if differentiation into neurons was occurring at a low rate. To determine if differentiation occurred at different rates into tegument versus neurons, we quantified the percentage of double^+^ cells over *dysf*^+^ or *pcda7*^+^ cells within the lateral regions after a 3-day chase. Newly differentiated F-*ara*-EdU^+^ cells were seen in 63 ± 15% of *dysf*^+^ cells, whereas co-occurrence with *pcda7*^+^ cells was only 36 ± 14%. These results suggest that differentiation into the tegument happens more rapidly than differentiation into neurons. Our successful application of combined labeling demonstrates that our protocol opens many possibilities for the study of stem cell heterogeneity and lineage commitment.

## Discussion

Regeneration of anatomical structures or even whole bodies has been observed in species across many metazoan phyla [29]. Newly regenerated structures require a cellular source and can be derived from resident stem cells, dedifferentiation, or transdifferentiation [30]. In the case of the free-living planarian *S. mediterranea*, pluripotent stem cells are responsible for whole-body regeneration. Remarkably, transplantation of a single pluripotent neoblast can rescue lethally irradiated planarians from certain death [31]. While extensive research has gone into the study of stem cells and regeneration in planarians, their parasitic cousins have lagged behind. There is now an ever-growing body of research exploring the importance of stem cells in blood and liver flukes [15, 32], as well as tapeworms [13, 14, 33, 34]. In *H. diminuta*, cycling adult stem cells are responsible for their rapid growth and regenerative ability [7, 35]. Competence to regenerate is limited to the GR, and extrinsic signals within the GR are required for persistent regeneration. *H. diminuta* affords us the opportunity to address important questions about stem cells and regenerative ability among helminths. For example, (i) How many subpopulations of stem cells are there and is there a stably maintained pluripotent population? (ii) What is the plasticity of stem cell lineage commitment? (iii) What are critical branch points for differentiation trajectories? (iv) How do self-renewal and differentiation dynamics respond to external stimuli? These and many other outstanding questions can be addressed using *H. diminuta* as a model tapeworm with the right tools.

Like other flatworms, the germinative cells/stem cells appear to be the only dividing population in tapeworms [12]. However, this population is hardly homogeneous. In different tapeworm species, pan-stem-cell marker genes have been identified including *histone h2b* [7, 12], *polo-like kinase 1* [36], a long non-coding RNA known as *terminal-repeat retrotransposons in miniature* [37], *cellular inhibitor of PP2A* [38], and *laminB receptor* [7] to name a few. Stem cell subpopulations can also be molecularly defined and are typically associated with lineage commitment [12, 14, 38]. Whether or not a pluripotent subpopulation exists is less clear. We have shown that regeneration of proglottids only occurs from the GR and is dependent on resident stem cells. However, by transplanting cells from non-regenerating tissues into the GR of lethally irradiated tapeworms, proglottid regeneration can be rescued implying that one or more populations of stem cells from non-regenerating tissues are collectively pluripotent, given the right microenvironmental signals [7]. Hence, understanding the molecular heterogeneity of tapeworm stem cells and how they contribute to regeneration is still an underexplored field.

With the advent of single-cell sequencing technologies, we are gaining ever-increasing power to uncover markers of stem cells and stem cell subpopulations as well as their progeny [39, 40]. Transcriptional trajectories can also be used to hypothesize the stepwise gene expression changes that allow stem cells to differentiate into various tissue types. The ability to investigate how candidate stem cell genes are expressed *in situ* will be greatly aided by our ability to simultaneously detect candidate mRNA expression and label cells that are actively proliferating. In this study, we have optimized a technique that allows us to observe the spatial expression of genes along with proliferating cells. We also show that this protocol is flexible with regard to permeabilization strategies. This implies that F-*ara*-EdU and/or FISH can likely be combined with antibody staining as well. As the range of techniques expands, we will be able to uncover transcriptionally distinct subpopulations within the heterogeneous cycling stem cell compartment.

The strength of using F-*ara*-Edu instead of other thymidine analogs is that it is highly sensitive and has low toxicity [18]. In *H. diminuta*, we have found that doses as low as 10 nM F-*ara*-EdU can still be detected. Tapeworms pulsed with F-*ara*-EdU can also be cultured *in vitro* for at least 12 days, likely longer, with no discernable deleterious effects on worm health. Thus, bulk lineage tracing with F-*ara*-EdU can be customized to answer a variety of biologically significant questions. Here we demonstrate the utility of our protocol for bulk lineage tracing into two differentiated tissues: nerves and tegument. We find that F-*ara*-EdU only co-occurs with *pcda7* (nerves) and *dysf* (tegument) after a 3-day chase, indicating that the co-labeled cells were newly produced from stem cells that incorporated F-*ara*-EdU during the pulse period (Figs 5 and 6). Fewer double-positive cells were detected with *pcda7* compared to *dysf* (Fig. 6B–D). This could be due to the preponderance of tegumental cells compared to neurons. It could also be due to different rates of differentiation. By reversing the reference population, we find that more F-*ara*-EdU^+^ stem cells differentiate into *dysf*^+^ cells.

Our method complements recent advances in bulk lineage tracing within larval stages of other tapeworm species such as *Echinococcus multilocularis* [38] and *H. microstoma* [14]. The tapeworm research community is becoming well poised to test hypotheses about differentiation dynamics and uncover transcriptional changes associated with lineage commitment or multi/pluripotency. In the absence of widely applicable transgenesis to perform true lineage tracing, combinatorial staining procedures are extremely useful. Future optimization may also allow us to incorporate additional thymidine analogs such as bromodeoxyuridine, which effectively labels planarian and tapeworm stem cells [41]. This would open the possibility of exploring gene expression and stem cell dynamics during self-renewal versus differentiation as has been shown in blood flukes [15, 42].

Understanding germ cell development in helminths has important implications for parasite transmission and even disease progression. In the tapeworm GR, the germline and somatic fates are likely segregating but we do not know where, how, and what regulates these transitions. Are tapeworms capable of regenerating and respecifying germ cells or must germ cells be generated from an inexhaustible population of germline stem cells? Once germline markers are uncovered, the methods in this study will help elucidate the self-renewal and differentiation dynamics of germline versus somatic fate during growth and regeneration.

A weakness of our study is that we have only performed combinatorial staining with riboprobes against four unique transcripts. While our success suggests that this protocol will likely be applicable to many genes of interest, further optimization might be necessary for transcripts expressed at low levels, in intractable cell types, or at transient cell states. Making this detailed protocol widely available will enable other researchers to apply this technique to their own research questions. This technique will likely be widely used in studies with *H. diminuta* as it opens our ability to address how stem cells contribute to development, growth, regeneration, reproduction, and interactions at host–parasite interfaces.

## Supplementary Material

bpaf011_Supplementary_Data

## Data Availability

All data are incorporated into the article. Raw data and plasmids will be shared upon request to the corresponding author.
