# Supplementary material for: Combined fluorescent in situ hybridization and F-ara-EdU staining on whole mount Hymenolepis diminuta
Source: Biol Methods Protoc. 2025 Feb 13;10(1):bpaf011. doi: 10.1093/biomethods/bpaf011 (PMC11886792; doi:10.1093/biomethods/bpaf011)
Supplement: bpaf011_Supplementary_Data [file bpaf011_supplementary_data.zip › Supp Table 1.pdf]

Description	Source	Part number
**Solutions, chemicals and kits**		
Hank’s Balanced Salt Solution (HBSS)	Corning	MT21023CV
antibiotic-antimycotic	Fisher	15240062
2’-deoxy-2’-fluoro-5-ethynyluridine (F-*ara*-EdU)	Sigma-Aldrich	T511293
dimethyl sulfoxide (DMSO)- for staining	Sigma-Aldrich	D2650
DMSO Hybri-Max- for use on tapeworms including for diluting F-*ara*-EdU	Sigma-Aldrich	D2650-5X5ML
defibrinated sheep blood	Hemostat	DSB500
BD Difco nutrient agar	Fisher	BD 213000
7.5% sodium bicarbonate	Corning	25035CI
diethyl pyrocarbonate (DEPC)	Sigma-Aldrich	D5758
proteinase K	Fisher	25530049
triethanolamine hydrochloride (TEA)	Sigma-Aldrich	T1502
acetic anhydride	Sigma-Aldrich	320102
yeast RNA	Invitrogen	AM7118
deionized formamide	Invitrogen	AM9342
dextran sulfate	Sigma-Aldrich	D8906
Roche Western Blocking Reagent (RWBR)	Sigma-Aldrich	11921681001
DyLight633 NHS ester (DL-633)	Pierce	46400
5-(and-6)-carboxytetramethylrhodamine (TAMRA)	Invitrogen	46410
5/6 carboxyfluorescein (FAM)	Pierce	C-1171
tyramine hydrochloride	Sigma-Aldrich	T2879
N-, N-dimethylformamide (DMF)	Sigma-Aldrich	D4551
triethylamine	Sigma-Aldrich	T0886
sodium azide	Sigma-Aldrich	S2002
Oregon Green 488 azide	Fisher	O10180
ascorbic acid	Sigma-Aldrich	A7631
horse serum	Sigma-Aldrich	H1270
fish gelatin	Sigma-Aldrich	G7765
4′,6-diamidine-2′-phenylindole dihydrochloride (DAPI)	Sigma-Aldrich	D9542
Zymo DNA clean and concentrator-5 kit	Genesee	11-303
RNAse inhibitor	Promega	N2515
DIG-11-UTP	Roche (Sigma-Aldrich)	11209256910
100 mM rNTPs	Invitrogen	PRE6000
spermidine	Sigma-Aldrich	S0266-1G
DTT (DL-Dithiothreitol)	Sigma-Aldrich	10197777001
RQ1 RNAse-free DNAse	Promega	M6101
**Antibodies**		
anti-digoxigenin conjugate to peroxidase domain, Fab fragments (anti-DIG-POD)	Roche	11207733910
anti-Oregon Green 488-HRP antibodies	Invitrogen	A-21253

**Equipment**		
16-G 4” curved 3mm ball gavage needles	Braintree Sci.	N-PK 009
Hamilton blunt needle (Luer Lock)	Fisher	14-815-601
stainless steel hooks	Moody Tools (Amazon)	55-0289
100 μm mesh bottoms (medium)	CEM	12440
Jaece identi-plug gas permeable stoppers	Fisher	14-127-40B

Supplemental Table 1: Compilation of reagents and sources used in this study.
