# Supplementary material for: Combined fluorescent in situ hybridization and F-ara-EdU staining on whole mount Hymenolepis diminuta
Source: Biol Methods Protoc. 2025 Feb 13;10(1):bpaf011. doi: 10.1093/biomethods/bpaf011 (PMC11886792; doi:10.1093/biomethods/bpaf011)
Supplement: bpaf011_Supplementary_Data [file bpaf011_supplementary_data.zip › Supp Figure 1.pdf]

***Hymenolepis diminuta (H. diminuta)*** **infection into rats**

1. Clean the surgical station using 70% ethanol.
2. In a 100 mm petri dish lid, dissect the *Tenebrio molitor* beetles that have been infected for at least 1 month with *H. diminuta* by first removing the head and snapping off the legs from the base of the beetle.
3. Position the beetle's body in the corner of the bottom half of a 100 mm, 0.5% BSA-coated petri dish, and add a few squirts of 0.85% NaCl.
4. Thoroughly dissect the beetle using straight forceps, tearing apart muscles and different segments to dislodge any cysticercoids.
5. Using a 0.5% BSA-coated tip, transfer the cysticercoids into a new 60 mm BSA-coated petri dish with fresh 0.85% NaCl. Repeat this process 2-3 times to obtain a clean cysticercoids suspension.
6. Aspirate cysticercoids (100-350 on average per rat) into a BSA-coated gavage needle with a ball tip, ensuring that the total volume in the syringe does not exceed 0.5 mL. *Note: Crowding effects have been reported when H. diminuta infections were performed at high densities. The most significant growth defects were observed 7 days post-infection or later although reduced number of proglottids were observed 5-6 days post infection [1,2]. In our experience, at 6 days post-infection, the variation in worm lengths within batches is comparable regardless of infection size. 6-day-old worms from over 400 cysticercoid infections are still competent to regenerate in vitro. We maximize the infection sizes to reduce the number of rats sacrificed per week. Obtaining 6-day-old worms also allows us to capture reproductive proglottids that would not have formed at earlier timepoints. However, for the purpose of maintaining the lifecycle, tapeworms*

*must be grown to full reproductive maturity. In such cases, we limit the infection size to 10 cysticercoids or less.*

7. Gently but firmly hold the rat, insert the needle into the rat's mouth, dispense the cysticercoid suspension fully, and then remove the needle.
8. Place the rat back in its cage until the desired date for harvesting *H. diminuta* (typically 6 days later).

***H. diminuta* harvest**

1. Sterilize the surgical station using 70% ethanol.
2. Prepare the station by laying out an absorbent pad, Hank's Balanced Salt Solution (HBSS), 250 mL and 600 mL beakers, a 60 mL syringe with a blunt needle, and dissection scissors. Fill the 250 mL beaker with approximately 100 mL of HBSS solution. Extract 60 mL with the syringe and needle, setting it aside while saving any excess HBSS.
3. Humanely euthanize rats carrying 6-day-old *H. diminuta* with CO2, followed by cervical dislocation to ensure secondary euthanization.
4. Transfer the euthanized rat to the prepared surgical station and use dissecting scissors to incise the abdominal cavity until the sternum is reached.
5. Locate the stomach beneath the sternum and cut the small intestine connected to the stomach.
6. Once the upper portion of the small intestine is separated from the stomach, carefully extend it until the large intestine is visible. Cut the base of the small intestine where it connects to the large intestine.

*Note: Tapeworms will be present in the last ⅓ of the small intestine (ileum); therefore, ensure that the cut is made at the junction with the large intestine to avoid missing worms.*

7. Insert the needle of the filled 60 mL syringe containing HBSS media into the initial part of the small intestine. Ensure a secure grip between the small intestine and the needle to prevent detachment when delivering the liquid.
8. Gradually dispense HBSS media through the small intestine to expel the feces.
9. Cease liquid dispensing just before it reaches the outlet, allowing the liquid pressure to force and flush out the feces into the 600 mL beaker.
10. After complete expulsion of liquid and feces, compress the intestine to ensure all contents are collected in the beaker. Swirl the mixture and add the remaining HBSS from the 250 mL beaker.
11. Disconnect the needle, wash it along with the scissors using water, and disinfect both instruments using 70% ethanol. Spray the surfaces of the instruments and the inside of the needle. Discard the syringe in the sharps bucket and sanitize the surgical station. Dispose of the rat following institutional guidelines.

**F-*ara*-EdU Treatment**

1. Clean the worms by transferring them into fresh Working Hanks 4 (WH4; HBSS with antibiotic-antimycotic and 4 g/L glucose) several times and move them through the air-water interface with a stainless steel hook.
2. Perform F-*ara*-EdU uptake soaking worms in 0.1 µM F-*ara* EdU/ 1% DMSO/WH4 at 37°C for a duration of 1 hour.

*Note: The stock F-ara-EdU solution, with a concentration of 10 µM, is prepared by dissolving it in tissue culture grade dimethyl sulfoxide (DMSO) from frozen aliquots. It is crucial to perform a 1:100 dilution from the 10 µM F-ara EdU stock, ensuring a final DMSO concentration of 1%.*

**For Pulse Experiment**

1. Wash the worms in WH4 three times before immediate fixation.

**For Pulse-Chase Experiment**

1. Prepare biphasic cultures with both solid and liquid phases.
2. Prepare the solid phase by mixing 30% heat-inactivated defibrinated sheep blood with 70% blood agar base (22.9 g/L BD Difco nutrient agar, 5 g/L NaCl).
3. Distribute 10 mL of the mixture into 50 mL Erlenmeyer flasks and allow it to solidify.
4. Add 10 mL of WH4 media to the solidified agar in each flask and top with a gas permeable stopper.
5. Preincubate the flasks in a hypoxic incubator (5% O2, 5% CO2, 90% N2) for 1 day.
6. Adjust the pH of the liquid phase to pH7.2-7.5 by adding 300 µL of 7.5% sodium bicarbonate.
7. Use a stainless-steel hook to transfer the worms into the liquid phase and incubate the worms in the liquid phase for 3 days under the same hypoxic conditions.
8. After 3 days, clean the worms by washing them in fresh WH4 media.
9. Fix the worms after washing for downstream analyses.

**Fixation**

1. Wash the samples three times with HBSS over a total period of 10-15 minutes.

2. Transfer clean worms into a glass scintillation vial containing approximately 10 mL of HBSS, avoiding overcrowding.

   *Note: Limit the number of 6-day-old worms to 20 to prevent overcrowding.*

3. Use a hot plate to heat autoclaved DI water in a beaker to 80°C.
4. Discard the HBSS and replace it with approximately 3-6 mL of 80°C DI-water (2 swigs with a transfer pipette). Swirl immediately to extend the worms. Quickly discard the water and replace it with 10 mL of 4% FND fixative (4% formaldehyde/10% DMSO/1% NP40/PBSTx-DEPC [1X Phosphate Buffered Saline with 0.3% Triton X-100 pretreated with 0.1 M diethyl pyrocarbonate]).
5. Fix the worms for 30 minutes at room temperature, being careful not to over-fix.
6. Wash the worms three times with 10 mL of PBSTx-DEPC for 5 minutes each.
7. To dehydrate the worms in methanol, perform one wash with 50% methanol/PBSTx-DEPC and three washes with 100% methanol, each lasting 5 minutes.

   *Note: For storage, fill the vial almost to the brim with 100% methanol and store it at -20°C. Keep it stored for at least 2 days before use.*

**In situ hybridization**

1. Rehydrate samples gradually by leaving approximately 5 mL of 100% methanol and then adding 5 mL of 50% methanol/PBSTx-DEPC for 5 minutes.
2. Perform washes with 50% methanol/PBSTx-DEPC for 5 minutes, followed by 25% methanol/PBSTx-DEPC for 5 minutes, and wash with PBSTx-DEPC three times for 5 minutes each.
3. Replace the solution with 10 mL of Proteinase K solution (10 µg/mL Proteinase K, 0.1% SDS in PBSTx-DEPC) and gently agitate for approximately 30 minutes.

*Note: Do not use a shaker, as it can break the tissues.*

4. Wash twice with 10 mL of 0.1 M Triethanolamine hydrochloride (TEA)-DEPC for 5 minutes each, then replace with a fresh 10 mL of 0.1 M TEA-DEPC.
5. Add 25 μL of acetic anhydride directly in a fume hood, swirling to ensure the acetic anhydride dissolves completely. After 5 minutes, add another 25 μL of acetic anhydride and leave it for an additional 5 minutes.

   *Note: Since acetic anhydride is sensitive to water vapor, pipette it only right before use.*
6. Wash twice with 10 mL PBSTx-DEPC for 5 minutes each.

   *Note: Acetic anhydride is hazardous. Ensure proper disposal.*
7. Post-fix in 4% formaldehyde/PBSTx-DEPC for 10 minutes at room temperature.
8. Wash three times with 10 mL of PBSTx-DEPC for 5 minutes each.
9. Transfer worms to medium incubation baskets with 100 μm mesh bottoms in a 24-well plate. Replace the solution with equal parts PBSTx-DEPC and prehybridization buffer (50% deionized formamide/5X SSC [Simple Sodium Citrate from 20X stock of 0.3 M sodium citrate, 3 M sodium chloride], 0.1 mg/mL yeast RNA, 1% Tween-20 in DEPC-treated water) for 10 minutes.
10. Replace with 800 μL of prehybridization buffer (prewarmed to 56°C) and incubate worms at 56°C for 2 hours.
11. Before the conclusion of the 2-hour prehybridization, prepare the transcript-specific riboprobe mix. Heat aliquots of each riboprobe stock in a thermocycler at 75-80°C for 10 minutes, then cool on ice. Dilute the riboprobes appropriately (typically 1:1000 from stocks of 50 ng/μL) in hybridization buffer (prehybridization buffer with 5% dextran sulfate, also prewarmed to 56°C).

12. Replace the prehybridization solution with the riboprobe mix and incubate at 56°C for 16 hours or overnight. For a weak signal, a 2-day incubation may be necessary.

    *Note: After riboprobe hybridization, DEPC treatment of solutions is no longer necessary since RNase activity is no longer a concern, as double stranded RNA is highly stable.*

13. Preheat 2X-SSCx and 0.2X-SSCx (20X SSC diluted and supplemented with 0.1% Triton X-100) to 56°C.
14. Remove half of the riboprobe mix and replace it with an equal volume of 2X-SSCx to equilibrate for 20 minutes.

    *Note: If possible, perform this step on a heat block at 56°C, or minimize the time spent at room temperature.*

15. Wash twice in 800 μL of 2X-SSCx at 56°C.
16. Perform four washes in 800 μL of 0.2X-SSCx at 56°C.
17. Move to room temperature and wash twice in 800 μL of TNTx (0.1 M Tris pH 7.5, 0.15 M NaCl, 0.3% Triton X-100) for 10 minutes each.
18. Block in 800 μL of blocking solution (10% horse serum, 0.5% RWBR [Roche Western Blocking Reagent] in TNTx) for 2 hours at room temperature.
19. Remove the blocking solution and incubate with the antibody solution (anti-DIG-POD antibodies at 1:2000 in blocking solution) overnight at 4°C.
20. Remove the antibody solution and wash the worms for 5 minutes, then for 10 minutes, followed by six washes for 20 minutes each in 800 μL TNTx.
21. Replace TNTx with 800 μL tyramide signal amplification (TSA) solution (1:250 DyLight633-tyramide [made in-house from NHS ester (Pierce: 46400)]/1:1000 4-IPBA [from 20 mg/mL stock of 4-iodophenylboronic acid (Sigma: 471933)], 0.003% H2O2,

TSA buffer [2 M sodium chloride, 100 mM borate pH 8.5]) and perform the TSA reaction for 20 minutes.

*Note: In-house tyramide-fluorophore conjugates were synthesized according to Ryan King's adaptation of (Hopman et al., 1998), previously published in King and Newmark, 2013.*

22. Wash four times in TNTx for 10 minutes each.
23. Inactivate peroxidase activity by incubating in 100 mM sodium azide in PBSTx for 45 minutes.

**<u>F-*ara*-EdU staining of *H. diminuta*.</u>**

1. Wash peroxidase-inactivated samples in TNTx four times for 10 minutes each.
2. Treat with Proteinase K solution for 30 minutes at room temperature without shaking until the desired permeability is reached. Periodically agitate the vials.

   *Note: A minimum 15-minute incubation is required.*
3. Post-fix in 4% formaldehyde/PBSTx for 10 minutes at room temperature.
4. Wash three times in PBSTx for 5 minutes each.

   *Note: If using medium incubation baskets, perform the transfer after one wash, and then continue washing in the baskets. Small worms can be processed whole, while larger worms should be cut into smaller pieces.*

   *Optional: Incubate in NDP permeabilization solution (1% NP40/10% DMSO/PBSTx) for 1 hour at room temperature, followed by three washes in PBSTx if increased permeabilization is warranted.*
5. For the Click-iT reaction, worms can be placed in 1.7 mL Eppendorf tubes to minimize the reaction volume. Remove as much PBSTx as possible (under the microscope) and

add 200 µL Click-iT reaction mix (0.1 M PBS, 1 mM CuSO4, 0.1 mM Oregon Green 488 azide, 100 mM ascorbic acid, added in that order with mixing in between).

*Note: This can also be done in baskets. It is important to scale up the reaction volume according to the amount of tissue; in baskets, 300 µL is an absolute minimum.*

6. Incubate worms for 30 minutes at room temperature.

   *Note: For Eppendorf tubes, flick to mix periodically. For baskets, do not use the orbital shaker, as the 24-well plate needs to be flat. Periodically rotate the plates on a flat surface.*

7. Wash three times in PBSTx.

   *Note: For Eppendorf tubes, transfer to baskets during the first wash.*

8. Block in K-block (5% heat inactivated horse serum, 0.45% fish gelatin, 0.3% Triton X, 0.05% Tween-20 in 0.1 M PBS) at room temperature for 2 hours.

   *Note: Blocking can be done overnight at 4°C. K-block is versatile and effective for many different antibodies but other blocks are likely also sufficient.*

9. Add anti-Oregon Green 488-HRP antibody at 1:1000 in K-block and incubate overnight at 4°C.

10. Wash eight times in TNTx (5 minutes for the first wash, 10 minutes for the second wash, and then 20 minutes each for the remaining six washs).

11. Perform the TSA reaction as described above, but with 1:500 TAMRA-tyramide (made in-house from 5- (and -6)- carboxytetramethylrhodamine) for 20 minutes.

12. Wash worms three times for 10 minutes each in TNTx.

13. Stain nuclei in 1 µg/mL 4′,6-Diamidine-2′-phenylindole dihydrochloride (DAPI)/TNTx overnight at 4°C.

14. Wash three times for 5 minutes each in TNTx.

    *Note: If samples are too sticky and fragile, they can be post-fixed and washed.*

15. Remove worms from baskets and equilibrate in mounting solution (80% glycerol, 10 mM Tris pH 7.5, 1 mM EDTA) for at least one night at room temperature.

16. Transfer worms to a clean slide and remove excess mounting solution. Cover with a coverslip with little clay feet at the edges. Apply gentle pressure to compress the worms, add mounting solution at the edge of the slide to fill the area, and seal with nail polish.

**Imaging**

1. Capture single-plane micrographs or z-stacks using a 63x objective (Plan-Apochromat 63x/1.40 Oil DIC M27) on the confocal microscope with plane thickness= 0.35 μm.
2. For maximum intensity projections, capture z-stacks using a 20x objective (Plan-Apochromat 20x/0.8 M27, FWD=0.55 mm) with plane thickness= 2 μm or less.
3. Perform all imaging on a Zeiss LSM 900 laser scanning confocal microscope or equivalent microscope.

**References**


1. Roberts, L. S. The influence of population density on patterns and physiology of growth in *Hymenolepis diminuta* (Cestoda:Cyclophyllidea) in the definitive host. *Experimental parasitology*. 1961;11, 332–371.

2. Bolla, R. I. and Roberts, L. S. Developmental physiology of cestodes—X. The effect of crowding on carbohydrate levels and on RNA, DNA and protein synthesis in *Hymenolepis diminuta*. *Comp. Biochem. Physiol. Part A: Physiol.* 1971;40, 777–787.
